# Deaths from Chloroform

**Published:** 1892-06

**Authors:** 


### DEATHS FROM CHLOROFORM.

During the past month the daily press of this city has given publicity to at least two cases of death attributable, or attributed to chloroform. One occurred during childbirth, and the coroner's autopsy showed a fatty heart.

The other took place in a man of some fifty years, who was being anæthetized for a finger amputation.

It is not our purpose to discuss here the relative merits of the various anæsthetics, but to call attention to two recent propositions, which seem at once sensible and timely.

The first is an inquiry (*British Medical Journal*, note in *New York Medical Journal*) on a large scale which is to be made in the hospitals throughout Great Britain during the year 1892 regarding anæsthetics. "Eminent surgeons, anæsthetists, and general practitioners will contribute their clinical experience, as supplemental to the conflicting results obtained by the experimental workers. The research will be made under the auspices of the British Medical Association. An influential and fairly constituted committee has charge of the plan of the inquiry, and record books have been prepared for the use of those who are willing to co-operate. These books have been carefully drawn up so as to secure uniformity on the part of the reporters, and they contain full instructions." Mr. Jonathan Hutchinson heads the committee, which contains the names of many of the best known men among the English surgeons.

A somewhat similar proposition comes from Braatz, one of the assistants at the Heidelberg clinic, who, while reviewing a paper by Djaknow, on the "Explanation of the Clinical Evidences Accompanying Death from Chloroform,"* calls attention to the fact that, no less than seven years ago,† he proposed that permanent chloroform committees be instituted, whose duty it should be to carefully examine into and report upon all deaths attributable to an anaesthetic. It goes without saying, that these committees should be made up of men prominent in their own communities. They would report at stated intervals, and their proceedings could find publication in a designated periodical.

Such a work of investigation could, in this country, be very well undertaken by committees appointed by such a representative body as the American Surgical Association.

There could be a sub-committee composed of, let us say, three men in each center of considerable size, whose duty it should be to make careful scientific investigation of each death attributed to an anæsthetic, occurring in its territory.

These sub-committees could in turn report to the central committee, which latter body should, as said, publish its conclusions at stated intervals.—*N. Y. Med. Record.*

*Centralblatt fur Chirurgie, 1891, p. 894.

†St. Petersburger Med. Wochenschrift, 1884.

